# Site 2 of the *Yersinia pestis* substrate-binding protein YfeA is a dynamic surface metal-binding site

**DOI:** 10.1107/S2053230X21008086

**Published:** 2021-08-24

**Authors:** Christopher D. Radka, Stephen G. Aller

**Affiliations:** a Department of Infectious Diseases, St Jude Children’s Research Hospital, Memphis, TN 38105, USA; b Department of Pharmacology and Toxicology, University of Alabama at Birmingham, Birmingham, AL 35294, USA

**Keywords:** *Yersinia pestis*, YfeA site 2, substrate-binding proteins, inter-protein metal coordination, crystallography, zinc, manganese, transition-metal homeostasis, plague

## Abstract

The substrate-binding protein YfeA (also known as YPO2439 or y1897) is a polyspecific metal-binding protein that is crucial for nutrient acquisition and virulence in *Yersinia pestis*, the causative microbe of plague. YfeA folds into a monomeric c-clamp like other substrate-binding proteins and has two metal-binding sites (sites 1 and 2). Site 2 is a bidentate surface site capable of binding Zn and Mn atoms and is a unique feature of YfeA. Occasionally, the site 2 residues of two YfeA molecules will cooperate with the histidine tag of a third YfeA molecule in coordinating the same metal and lead to metal-dependent crystallographic packing. Here, three crystal structures of YfeA are presented at 1.85, 2.05 and 2.25 Å resolution. A comparison of the structures reveals that the metal can be displaced at five different locations ranging from ∼4 to ∼16 Å away from the canonical site 2. These observations reveal different configurations of site 2 that enable cooperative metal binding and demonstrate how site 2 is dynamic and freely available for inter-protein metal coordination.

## Abstract 2

The *Yersinia pestis* substrate-binding protein YfeA contains two polyspecific metal-binding sites, and site 2 is capable of collaborating with other protein molecules for inter-protein metal coordination. The inter-protein metal coordination can occur by multiple coordination geometries and may be flexible enough to enable metal transfer to multiple metal-binding proteins.

## 1. Introduction   

Plague is an infection by *Yersinia pestis* that has caused several pandemics and reached all corners of the globe (Vogler *et al.*, 2013 ▸). Recent outbreaks in Africa (Respicio-Kingry *et al.*, 2016 ▸; Abedi *et al.*, 2018 ▸; Andrianaivoarimanana *et al.*, 2019 ▸; Randremanana *et al.*, 2019 ▸) and China (Shi *et al.*, 2018 ▸) show that plague remains a threat to public health. Transition-metal acquisition is a key process in plague pathogenesis. Disruption of the manganese and iron transporter Yfe renders *Y. pestis* avirulent by intravenous infection or severely compromises virulence on subcutaneous infection (Bearden & Perry, 1999 ▸). Yfe is an ATP-binding cassette transporter made up of the substrate-binding protein (SBP) YfeA, the ATP-binding protein YfeB and the membrane components YfeCDE (Bearden *et al.*, 1998 ▸).

**Figure 1:**
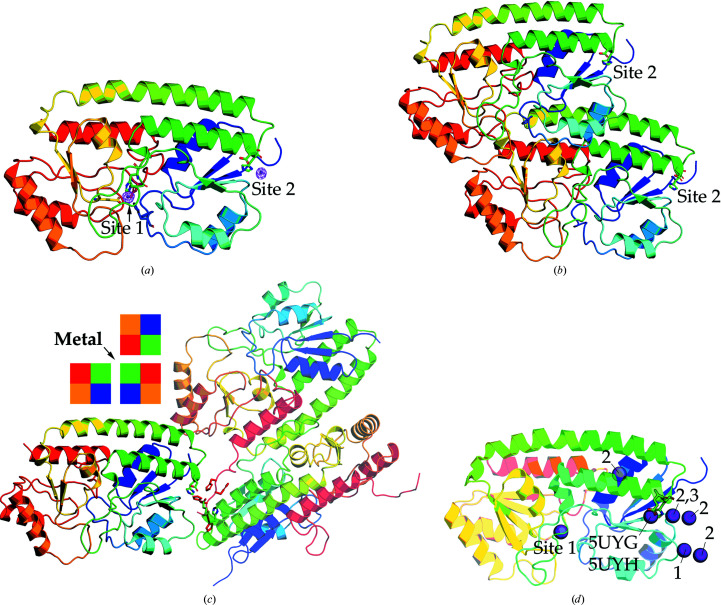
Arrangement of YfeA protomers and crystallographic packing. YfeA protomers are colored in a spectrum from blue (amino-terminus) to red (carboxy-terminus) and metals are shown as spheres. (*a*) Site 1 is a buried metal-binding site with four amino-acid ligands and site 2 is a surface site with two amino-acid ligands. Anomalous difference electron density contoured at 4σ (magenta mesh) shows the location of each metal. (*b*) Two YfeA protomers are present in the asymmetric unit of each crystal and the site 2 motifs are distant from each other. (*c*) Each protomer interacts with two symmetry-related protomers (shown as transparent molecules) to form a crystallographic complex. In the arrangement shown, two site 2 motifs (green) flank residues from a His_10_ tag (red). The protomers in the complex are related by rotation and reflection symmetry along the screw axis. Blocks colored according to the YfeA sequence show the symmetry relationship between protomers and how the metal is wedged between protomers. (*d*) Overview of the positional distribution of metals in the YfeA crystal packing. A metal is present in site 1 in all structures. PDB entries 5uyg and 5uyh show the canonical site 2 metal site. Metals present in YfeA site 2 inter-coordinated structure 1, structure 2 and structure 3 are denoted 1, 2 and 3, respectively. A common metal is present in structures 2 and 3.

The crystal structure of YfeA shows the prototypical SBP c-clamp fold with a primary substrate-binding site located in the c-clamp arch (site 1; Scheepers *et al.*, 2016 ▸). SBPs are classified into clusters according to their three-dimensional structures and the clusters correlate with the substrate specificity (Scheepers *et al.*, 2016 ▸). YfeA is a cluster A-I SBP that binds metal atoms such that site 1 binds Zn, Fe and Mn atoms and a secondary substrate-binding site located on the surface (site 2) binds Zn and Mn atoms (Fig. 1 ▸ *a*; Radka *et al.*, 2017 ▸). Both sites 1 and 2 are tetracoordinate, where four amino acids (His76/His141/Glu207/Asp282) coordinate the metal in site 1 and two amino acids (Glu162/His163) and two water molecules coordinate the metal in site 2 (Radka *et al.*, 2017 ▸). Multiple surface ancillary binding sites can be reversibly occupied by soaking YfeA crystals in zinc or back-soaking in metal-free mother liquor, but site 2 requires zinc or manganese to be present during crystallization and is not demetal­ated by back-soaking (Radka *et al.*, 2017 ▸). Furthermore, metal occupancy at site 2 influences which grouping of surface ancillary sites bind metal (Radka *et al.*, 2017 ▸). There is no clear path to shuttle substrate across the >26 Å distance between sites 1 and 2, and methods to generate apo YfeA (Radka *et al.*, 2018 ▸) and reconstitute site 1 metal-bound holo YfeA (Radka *et al.*, 2019 ▸) showed that site 2 does not play an apparent role in loading metal into site 1.

Here, structures in which metals are inter-coordinated by multiple YfeA molecules are reported at 1.85, 2.05 and 2.25 Å resolution. The crystal structures show how a metal can bounce around site 2 and trigger distinct assemblies of protein–protein interactions. The metal recruits site 2 residues, other surface residues and His_10_ tag residues from multiple protomers to collaborate in metal coordination and form crystal contacts in the lattice. These modes of engaging YfeA at site 2 presents the intriguing possibility that site 2 may participate in protein–protein interactions with other metal-binding proteins.

## 2. Experimental procedures   

### 2.1. Production and purification of YfeA   

Recombinant YfeA-His_10_ was overexpressed in *Escherichia coli* strain BL21(DE3) pLysS Singles (Novagen; catalog No. 70236) using the vector pET-22b and purified as described previously (Radka *et al.*, 2017 ▸). Briefly, YfeA-His_10_ was loaded onto a HisTrap HP column (GE Healthcare Life Sciences; catalog No. 17-5248-02), washed and eluted using a linear imidazole gradient from 0.02 to 1.0 *M* imidazole. Fractions containing the eluent were pooled and loaded onto a HiTrap Q HP column (GE Healthcare Life Sciences; catalog No. 17-1154-01), washed and eluted using a linear NaCl gradient from 0.0 to 1.0 *M* NaCl. Fractions containing the eluent were pooled and further purified by gel filtration on a HiLoad 26/600 Superdex 200 pg column (GE Healthcare Life Sciences; catalog No. 28-9893-36). The final purified protein was concentrated to 18 ± 5 mg ml^−1^ in 20 m*M* bis-Tris propane pH 6.3, 50 m*M* NaCl, 0.05%(*w*/*v*) NaN_3_ for crystallization.

### 2.2. Analysis of oligomeric state   

To determine whether MnCl_2_ triggered YfeA oligomerization, 5 mg ml^−1^ YfeA was incubated with and without 10 m*M* MnCl_2_ in 20 m*M* bis-Tris propane pH 6.3, 50 m*M* NaCl, 0.05%(*w*/*v*) NaN_3_ for 2.5 h at room temperature. The mixture was analyzed by gel filtration using a Superdex 200 10/300 GL column (Cytiva Life Sciences; catalog No. 17517501).

### 2.3. Crystallization, data collection and structure determination   

Crystals of YfeA-His_10_ were grown by the hanging-drop vapor-diffusion method at 20°C using a 1:1 ratio of 32%(*w*/*v*) PEG 4000, 20 m*M* bis-Tris propane pH 6.3, 50 m*M* NaCl, 0.05%(*w*/*v*) NaN_3_, 10 m*M* MnCl_2_ and 18 ± 5 mg ml^−1^ YfeA-His_10_. X-ray diffraction data were collected at the X-ray absorption-edge energy (*K* edge) for zinc or iron as described previously (Radka *et al.*, 2017 ▸). These energies were determined at the beamline for each data-collection session. Data collected near the Fe *K* edge can visualize both iron and manganese because the Fe *K*-edge energy is greater than the Mn *K*-edge energy. Collecting data at the lower Mn *K*-edge energy would visualize manganese but not iron or zinc. Iron and manganese are only weakly detected at the Zn *K* edge. The data were merged and scaled using *HKL*-2000 version 715 (Otwinowski & Minor, 1997 ▸). The structure was solved by molecular replacement using *Phaser* (Zwart *et al.*, 2008 ▸) as implemented in *Phenix* (Liebschner *et al.*, 2019 ▸) and monomeric YfeA (PDB entry 5uxs) was used as a search model (Radka *et al.*, 2017 ▸). Additional model building and structural refinement were performed using *Coot* (Emsley *et al.*, 2010 ▸) and *Phenix* (Liebschner *et al.*, 2019 ▸). The figures were generated using *PyMOL* (DeLano, 2002 ▸). Statistics for the X-ray data processing and model refinement are listed in Table 1 ▸. C^α^ r.m.s.d. calculations were performed using secondary-structure matching (SSM) Superpose in *Coot* (Emsley *et al.*, 2010 ▸).

## 3. Results   

Approximately 5% of the YfeA crystals grown in the presence of MnCl_2_ belonged to an orthorhombic space group with unit-cell dimensions *a* = 67.3 ± 0.2, *b* = 76.4 ± 0.6, *c* = 108.1 ± 1.9 Å (Table 1 ▸) and two YfeA protomers in the asymmetric unit (Fig. 1 ▸ *b*), whereas generally the YfeA crystals had one YfeA molecule in the asymmetric unit and belonged to an ortho­rhombic space group with unit-cell dimensions *a* = 41, *b* = 52, *c* = 113 Å or *a* = 62, *b* = 66, *c* = 66 Å (Radka *et al.*, 2017 ▸). Inspection of the arrangement of the YfeA protomers shows crystallographic packing such that site 2 of one protomer is in close proximity to site 2 from a second protomer and the His_10_ tag from a third protomer (Fig. 1 ▸ *c*). Structural alignment of each YfeA protomer from PDB entries 5uyg and 5uxs (monomeric YfeA with and without metal bound in site 2, respectively) yielded an r.m.s.d. of 0.54 ± 0.09 Å for 269–272 pairs of matched C^α^ atoms, indicating that the YfeA protomers here are highly similar to YfeA monomers. The r.m.s.d. among the present YfeA protomers is 0.47 ± 0.16 Å for 271–281 pairs of matched C^α^ atoms, indicating that the YfeA protomers are highly similar to one another. Some protomer pairs had more matched C^α^ atoms than others due to the resolution of additional residues in the carboxy-terminal His_10_ tag in some protomers. YfeA can also bind ancillary metals at additional surface sites (Fig. 1 ▸ *d*). These metals are coordinated by multiple protomers and enable crystal contacts in the lattice, but have incomplete coordination spheres due to missing solvent molecules or other nonprotein ligands (for example buffer molecules; Laitaoja *et al.*, 2013 ▸).

The site 2 substrate will be simply referred to as ‘the metal’ because site 2 binds zinc and manganese (Radka *et al.*, 2017 ▸), as also shown by the anomalous difference density from the diffraction data collected at the *K* edges (Section 2), and the coordination geometries in the crystal structures can apply to both.

**Figure 2:**
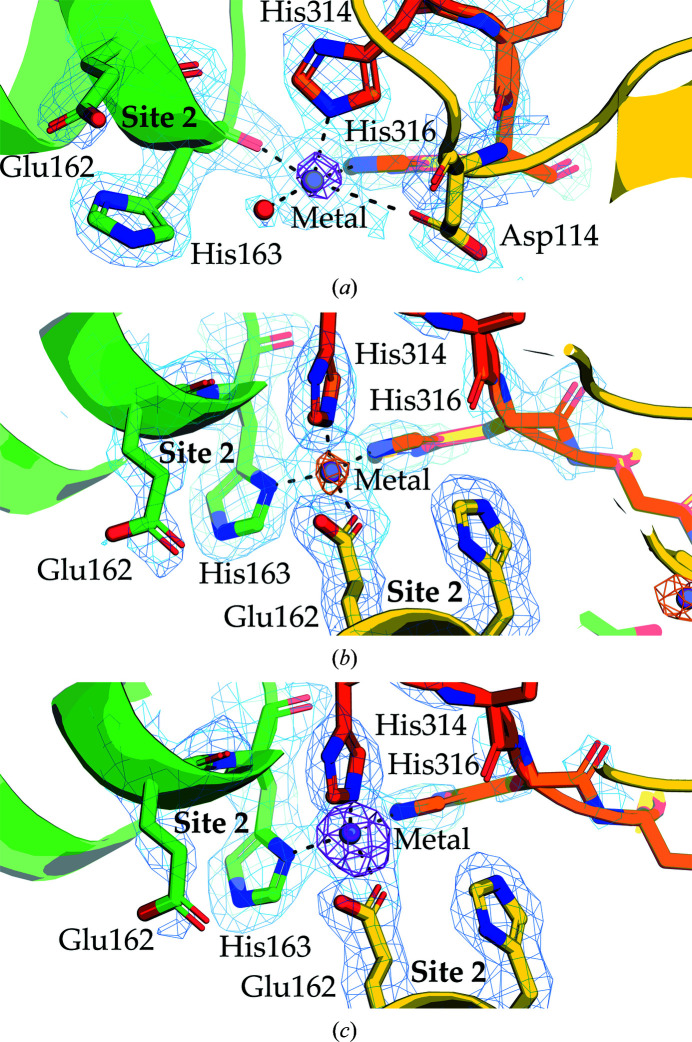
Enlarged views of YfeA metal-coordination complexes. Three YfeA protomers are colored green, yellow and orange. The YfeA models are overlaid with anomalous difference electron density contoured at 4σ (magenta or orange mesh) and electron density calculated from a 2*mF*_o_ − *DF*_c_ map contoured at 1σ (blue mesh). (*a*) YfeA site 2 inter-coordinated structure 1 contains square bipyramidal geometry at site 2. (*b*) YfeA site 2 inter-coordinated structure 2 contains tetrahedral geometry at site 2. (*c*) YfeA site 2 inter-coordinated structure 3 contains tetrahedral geometry at site 2.

In YfeA site 2 inter-coordinated structure 1, the metal is coordinated by the backbone carbonyl of site 2 residue His163 of one protomer, the side chain of surface residue Asp114 of a second protomer, His_10_-tag residues His314 and His316 of a third protomer and a water molecule (Fig. 2 ▸ *a*). Three histidine residues of the His_10_ tag are resolved in one protomer and no histidine residues are resolved in the other protomer in the asymmetric unit. The arrangement of ligands results in square bipyramidal coordination geometry, where the His314 ligand is at the apex of the pyramid and the other four ligands form the square base. Square pyramidal coordination geometry is commonly observed in protein–manganese (Udayalaxmi *et al.*, 2020 ▸) and protein–zinc (Laitaoja *et al.*, 2013 ▸) complexes.

In YfeA site 2 inter-coordinated structure 2, the metal is coordinated by the side chain of site 2 residue His163 of one protomer, the side chain of site 2 residue Glu162 of a second protomer and His_10_-tag residues His314 and His316 of a third protomer (Fig. 2 ▸ *b*). Therefore, two site 2 motifs participate in metal coordination. Five histidine residues of the His_10_ tag are resolved in one protomer and no histidine residues are resolved in the other protomer in the asymmetric unit. The four ligands form bond angles of ∼110–115° in this arrangement, which is consistent with tetrahedral coordination geometry. Tetrahedral coordination geometry is widespread in protein–transition metal complexes and is used at site 1 to coordinate zinc, manganese and iron (Radka *et al.*, 2017 ▸). Data for structure 2 were collected at the Fe *K* edge specifically because (i) Yfe is required for iron transport and full virulence of plague infection (Bearden & Perry (1999 ▸), (ii) the structure reveals unique metal binding that is not present in the other two structures and (iii) the X-ray energy confirms the presence of iron or manganese but cannot detect zinc anomalous scattering.

In YfeA site 2 inter-coordinated structure 3, the metal is coordinated by the same ligands as in structure 2 to achieve tetrahedral coordination geometry (Fig. 2 ▸ *c*). Similarly, five histidine residues of the His_10_ tag are resolved in one protomer and no histidine residues are resolved in the other protomer in the asymmetric unit. There are no ancillary metals present in the structure.

**Figure 3:**
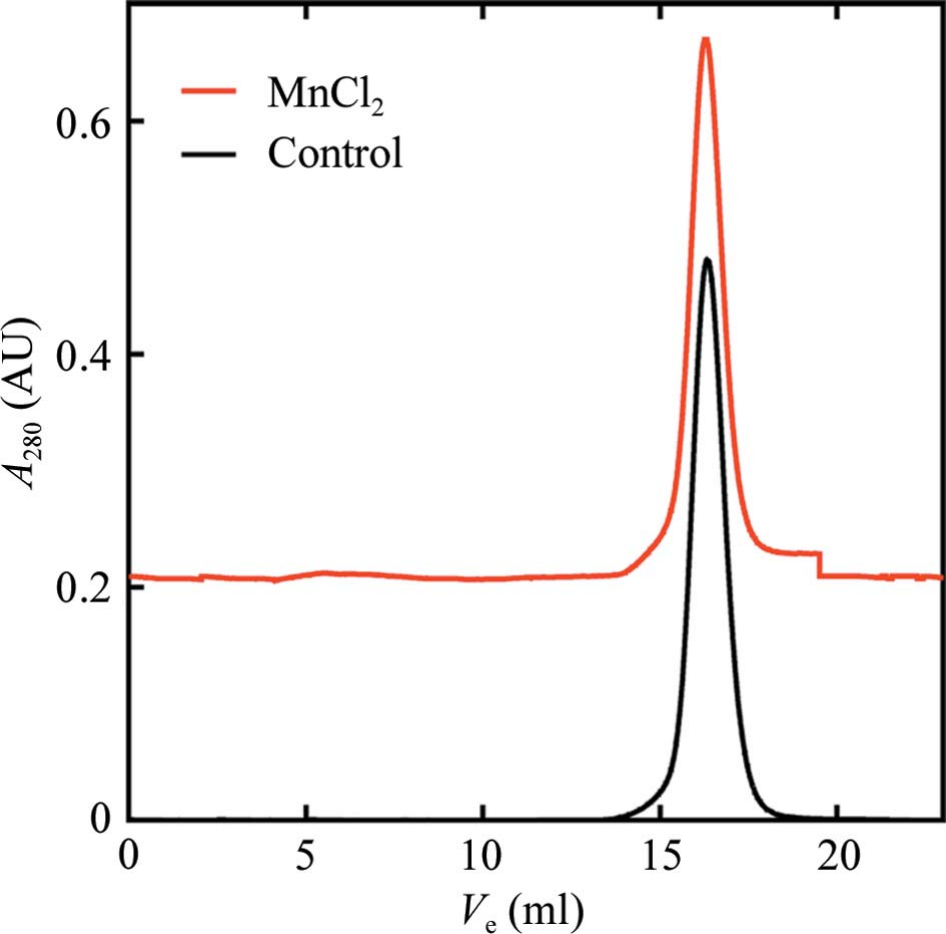
Gel-filtration profiles of YfeA-His_10_ in the presence or absence of MnCl_2_. Both peaks correspond to monomeric YfeA, indicating that oligomer formation does not occur under the conditions of the experiment.

Soluble YfeA was incubated in crystallization buffer with and without 10 m*M* MnCl_2_ for 2.5 h and the mixtures were seperated by gel filtration to determine whether MnCl_2_ triggered YfeA oligomerization in solution. The elution position under both conditions is consistent with monomeric YfeA, indicating that the perceived oligomerization is a property of crystallization (Fig. 3 ▸). The crystal-packing contacts are likely to stabilize the arrangement of protein molecules in the crystal lattice and the MnCl_2_ in the mother liquor enabled YfeA nucleation around site 2 in a metal-binding-dependent manner.

## 4. Discussion   

The bacterial ferritin FtnA contains a glutamate–histidine Fe-atom binding site that is the final observable stop (Fe_C_) in an internal translocation relay before iron is incorporated into a growing mineral for nutrient storage (Yao *et al.*, 2011 ▸). Proton transfer within the interior cavity is proposed as a key process to promote complementary ionization states of the binding residues with oxidation states of the iron for translocation relay (Yao *et al.*, 2011 ▸). Since the YfeA Glu–His site 2 is on the surface, the modulation of charge potential for substrate binding may instead come from bulk solvent and/or other surface elements.

**Figure 4:**
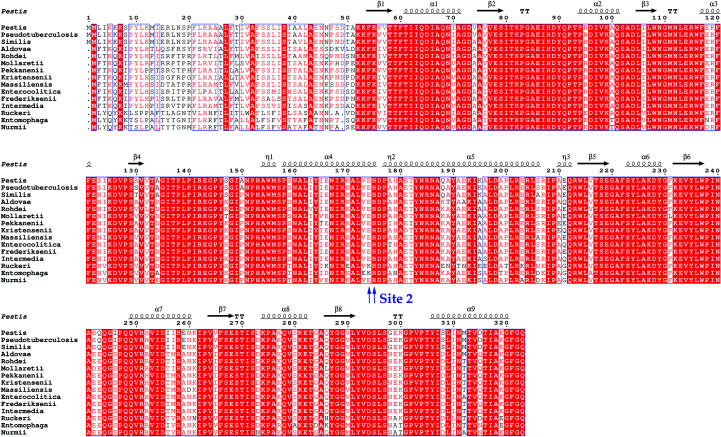
YfeA sequence analysis. YfeA secondary structure mapped onto a YfeA homolog sequence alignment across *Yersinia* species. The *Y. pestis* YfeA secondary-structure components (PDB entry 5uxs) are shown above a sequence alignment of *Y. pestis* (GenPept entry WP_002227896.1), *Y. pseudotuberculosis* (GenPept entry WP_072083356.1), *Y. similis* (GenPept entry WP_081761347.1), *Y. aldovae* (GenPept entry WP_049633611.1), *Y. rohdei* (GenPept entry WP_004713032.1), *Y. mollaretii* (GenPept entry WP_049677738.1), *Y. pekkanenii* (GenPept entry WP_049614033.1), *Y. kristensenii* (GenPept entry WP_050116424.1), *Y. massiliensis* (GenPept entry WP_019210183.1), *Y. enterocolitica* (GenPept entry WP_083158681.1), *Y. frederiksenii* (GenPept entry WP_087818068.1), *Y. intermedia* (GenPept entry WP_042569899.1), *Y. ruckeri* (GenPept entry WP_042525072.1), *Y. entomophaga* (GenPept entry WP_064518097.1) and *Y. nurmii* (GenPept entry WP_049600648.1). Site 2 amino-acid residues are identified by blue arrows under the alignment. Primary sequences of YfeA were aligned using *Clustal Omega* (https://www.ebi.ac.uk/Tools/msa/clustalo/) and the alignment was visualized using *ESPript* (http://endscript.ibcp.fr/ESPript/ESPript/index.php). Sequence numbering is that assigned to *Y. pestis* (GenPept entry WP_002227896.1).

**Figure 5:**
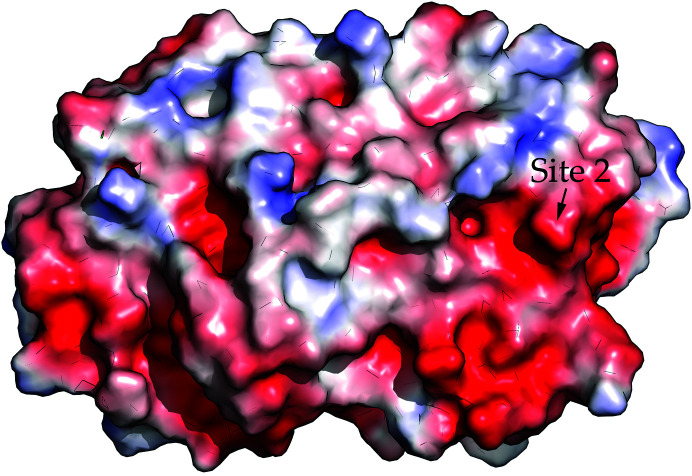
Site 2 is in an electronegative region of YfeA identified by a black arrow. The electrostatic surface potential of YfeA colored from red (−3 *kT* e^−1^) to blue (3 *kT* e^−1^) shows a distinct acidic patch surrounding site 2.

The amino-acid sequences of YfeA homologs from 15 unique *Yersinia* species were compared and strains of the environmental species *Y. entomophaga* (GenBank accession WP_064518097.1) and *Y. ruckeri* (GenBank accession AJI94945.1) were found to contain a site 2 lysine substitution for glutamate or a lysine insertion between the glutamate and histidine, but the histidine is conserved in all sequences (Fig. 4 ▸). The site 2 binding capacity has not been tested for these sequences, but should these homologs bind metal at site 2 then this would indicate that site 2 does not require a paired hydrogen-bond donor or acceptor like other SBP binding sites (Ledvina *et al.*, 1996 ▸). Instead, site 2 is in a region of negative electrostatic surface potential (Fig. 5 ▸). Binding sites with surface electrostatic potential often recruit substrates with complementary charge (Honig & Nicholls, 1995 ▸), and the electric field can modulate and enhance or attenuate metal binding (Dudev *et al.*, 2018 ▸). Therefore, the substrate selectivity and affinity of site 2 are likely to be influenced by favorable conditions that arise from the local polarization of an electronegative region and the availability of water molecules from bulk solvent that add to site 2 ligands to achieve tetracoordinate geometry.

Sites 1 and 2 have different local (for example electrostatic) and global (for example buried versus surface) environments that impact metal binding and possibly function. The c-clamp fold contributes all of the coordinate interactions at site 1, limiting metal binding to a single configuration in the buried site. On the other hand, protein and bulk solvent combine to achieve all of the coordinate interactions for the site 2 surface site. Nine YfeA ancillary metal-binding sites have previously been identified (Radka *et al.*, 2017 ▸) and three additional ancillary sites were identified in this work. These additional ancillary sites highlight multiple metal-binding sites in close proximity to site 2 that were not observed in previous work, and are jointly occupied in YfeA site 2 inter-coordinated structure 2 (Fig. 1 ▸ *d*). This metal-binding behavior is consistent with the characterization of YfeA as a zinc sink (Radka *et al.*, 2017 ▸) or a metal sponge. Back-soaking experiments have demonstrated that the metals deposited across the ancillary sites are loosely bound (Radka *et al.*, 2017 ▸) and may be scavenged by higher affinity proteins when metal abundance decreases. It is unclear whether the site 2-bound metal shares the same fate and/or purpose as the ancillary site-bound metal. Varied configurations of YfeA crystallographic packing show how multiple polypeptide chains can collaborate with site 2 to coordinate the metal. These configurations include a non-site-2 residue from the surrounding negative electrostatic region (YfeA site 2 inter-coordinated structure 1; Fig. 2 ▸ *a*) or multiple site 2 motifs (structures 2 and 3; Figs. 2 ▸ *b* and 2 ▸ *c*, respectively). Only one site 2 motif is needed to bind the metal, and the metal can be coordinated by backbone or side-chain ligands, making a potential metal handoff between proteins feasible. Participation of the YfeA His_10_ tag in metal coordination is intriguing because the zinc-binding SBP ZnuA has a histidine-rich loop near ZnuA site 1 that has been implicated in metal transfer to/from site 1 (Ilari *et al.*, 2011 ▸; Yatsunyk *et al.*, 2008 ▸; Hecel *et al.*, 2020 ▸). The participation of the YfeA His_10_ tag in collaborative metal coordination may show how a histidine-rich loop in ZnuA may slip in to scavenge the metal from YfeA site 2 and deliver it to ZnuA site 1.

Although a role for the YfeA His tag has not been seen in any of the previously published structures, the role here is somewhat artifactual but nevertheless still informative. Zinc coordination usually requires tetrahedral geometry with at least two histidine ligands. Site 2 of one YfeA molecule only contributes two of the four ligands, and only one is a histidine (His163). Therefore, His-tag zinc coordination shows that (i) the site 2 metal site is sufficiently close to the surface to participate in intermolecular metal coordination and (ii) the His-tag histidine is likely to mimic the metal coordination geometry that could occur with an as-yet-unidentified biological binding partner.

The possibility of metal transfer from YfeA site 2 to the Yfe transporter was considered and an alignment of YfeA against BtuF in complex with the Btu transporter (PDB entry 4fi3; Korkhov *et al.*, 2012 ▸) was performed. The alignment predicts that site 2 faces away from the translocation pathway of the transporter and is therefore unlikely to make meaningful contact with the YfeBCD transporter components that are equivalent to the Btu transporter components. YfeE is an additional transmembrane-protein component of the Yfe transporter for which a function has not been established and is a unique feature of the Yfe transporter (Bearden & Perry, 1999 ▸; Bearden *et al.*, 1998 ▸). There is no homology model for YfeE, which may also be a potential binding partner for site 2.

## Supplementary Material

PDB reference: YfeA oligomer crystal 1, form 1, 7me1

PDB reference: crystal 2, form 2, 7me2

PDB reference: crystal 3, form 2, 7me3

## Figures and Tables

**Table 1:** Data-collection and refinement statistics Values in parentheses are for the highest resolution shell.

	YfeA site 2 inter-coordinated structure 1	YfeA site 2 inter-coordinated structure 2	YfeA site 2 inter-coordinated structure 3
PDB code	7me1	7me2	7me3
Data collection			
Beamline	CMCF-BM, CLS	SER-CAT 22-ID, APS	SER-CAT 22-ID, APS
Wavelength (Å)	1.283 [Zn *K* edge]	1.74 [Fe *K* edge]	1.283 [Zn *K* edge]
Resolution range (Å)	46.10–2.05 (2.123–2.050)	45.71–1.85 (1.916–1.850)	41.72–2.25 (2.330–2.250)
Space group	*P*2_1_2_1_2_1_	*P*2_1_2_1_2_1_	*P*2_1_2_1_2_1_
*a*, *b*, *c* (Å)	67.5, 76.9, 110.3	67.3, 76.4, 107.4	67.1, 75.8, 106.6
α, β, γ (°)	90, 90, 90	90, 90, 90	90, 90, 90
Total reflections	68969 (4633)	89302 (6433)	52503 (5090)
Unique reflections	34743 (2408)	45565 (3472)	26354 (2553)
Multiplicity	2.0 (1.9)	2.0 (1.9)	2.0 (2.0)
Completeness (%)	94.53 (66.65)	94.93 (73.34)	99.54 (98.31)
Mean *I*/σ(*I*)	11.20 (7.13)	12.67 (3.22)	7.30 (4.32)
Wilson *B* factor (Å^2^)	21.27	17.57	14.75
*R*_merge_	0.043 (0.106)	0.031 (0.163)	0.081 (0.247)
*R*_meas_	0.062 (0.150)	0.044 (0.231)	0.114 (0.350)
*R*_p.i.m._	0.044 (0.106)	0.031 (0.163)	0.081 (0.247)
CC_1/2_	0.991 (0.904)	0.998 (0.920)	0.968 (0.654)
CC*	0.998 (0.975)	0.999 (0.979)	0.992 (0.889)
Refinement			
Reflections used in refinement	34731 (2406)	45563 (3472)	26344 (2552)
Reflections used for *R*_free_	1750 (127)	2129 (150)	1344 (112)
*R*_work_	0.166 (0.203)	0.152 (0.273)	0.171 (0.214)
*R*_free_ †	0.195 (0.256)	0.184 (0.303)	0.214 (0.265)
CC(work)	0.959 (0.893)	0.968 (0.904)	0.950 (0.810)
CC(free)	0.936 (0.833)	0.949 (0.785)	0.931 (0.831)
No. of non-H atoms			
Total	4870	4931	4637
Macromolecule	4327	4354	4354
Ligands (metal ions)	6	12	8
Solvent	554	609	292
No. of protein residues	552	554	557
R.m.s.d., bond lengths (Å)	0.005	0.008	0.007
R.m.s.d., angles (°)	0.69	0.91	0.90
Ramachandran favored (%)	98.17	97.64	97.27
Ramachandran allowed (%)	1.83	2.36	2.73
Ramachandran outliers (%)	0	0	0
Rotamer outliers (%)	0.43	0	0.64
Clashscore	3.01	3.67	4.36
Average *B* factor (Å^2^)			
Overall	29.15	25.35	22.96
Zinc	25.10	10.78	10.82
Macromolecules	28.45	24.22	22.74
Ligands	25.26	35.33	9.66
Solvent	34.67	33.23	26.61

† 5% of the data were reserved for calculating the *R*_free_ statistic.
